# Atomic Layer Deposition of Titanium Oxide-Based Films for Semiconductor Applications—Effects of Precursor and Operating Conditions

**DOI:** 10.3390/ma16165522

**Published:** 2023-08-08

**Authors:** Vladyslav Matkivskyi, Oskari Leiviskä, Sigurd Wenner, Hanchen Liu, Ville Vähänissi, Hele Savin, Marisa Di Sabatino, Gabriella Tranell

**Affiliations:** 1 Department of Materials Science and Engineering, Norwegian University of Science and Technology, Alfred Getz vei 2B, 7034 Trondheim, Norway; marisa.di.sabatino@ntnu.no (M.D.S.); gabriella.tranell@ntnu.no (G.T.); 2 Department of Electronics and Nanoengineering, Aalto University, Tietotie 3, 02150 Espoo, Finland; oskari.leiviska@aalto.fi (O.L.); hanchen.liu@aalto.fi (H.L.); ville.vahanissi@aalto.fi (V.V.); hele.savin@aalto.fi (H.S.); 3 SINTEF Industry, Høgskoleringen 5, 7034 Trondheim, Norway; sigurd.wenner@ntnu.no

**Keywords:** atomic layer deposition, titanium oxide, passivation films, ALD precursors

## Abstract

Two widely used atomic layer deposition precursors, Tetrakis (dimethylamido) titanium (TDMA-Ti) and titanium tetrachloride (TiCl_4_), were investigated for use in the deposition of TiOx-based thin films as a passivating contact material for solar cells. This study revealed that both precursors are suited to similar deposition temperatures (150 °C). Post-deposition annealing plays a major role in optimising the titanium oxide (TiO_x_) film passivation properties, improving minority carrier lifetime (τ_eff_) by more than 200 µs. Aluminium oxide deposited together with titanium oxide (AlO_y_/TiO_x_) reduced the sheet resistance by 40% compared with pure TiO_x_. It was also revealed that the passivation quality of the (AlO_y_/TiO_x_) stack depends on the precursor and ratio of AlO_y_ to TiO_x_ deposition cycles.

## 1. Introduction

Titanium oxide materials (TiO_x_) are used in a wide range of applications such as batteries [1], medicine [2], semiconductors [3], and solar photovoltaic (PV) cells [4,5]. Among the many metal oxides, titanium oxide has the closest band offset with c-Si [6], making it suitable for semiconductor/PV applications. In addition, the high thermal stability [6,7] and availability of deposition/formation methods [8,9,10] for TiO_x_ make its processing favourable.

In solar cell fabrication, titanium oxide first appeared as part of the anti-reflective coating [5] and is still used in protective coatings for solar cells [11]. Currently, the carrier selectiveness and passivation properties of TiO_x_ have gained interest in the semiconductor industry [12,13]. This is mainly due to the rapid market implementation of the TOPcon (passivated contact cell) [14] architecture, which has brought forward potential new passivation materials such as TiO_x_.

As a crystalline material, titanium dioxide has three different crystal polymorphs: rutile [15], brookite [16], and anatase [15]. Anatase and rutile are the most common in TiO_2_ fabrication, and the anatase phase is the most desirable for semiconductor applications because of its conducting and passivation properties [17]. The semiconductor industry has developed a range of techniques for TiO_x_ deposition such as atomic layer deposition (ALD) [18], E-beam electron evaporation [19], magnetron sputtering [20], and chemical vapour deposition [21], and the choice of deposition process affects the final quality of the TiO_x_ film [10].

ALD of titanium oxide has evolved as one of the main alternatives to sputtering, which is widely applied in the industry [22] and is under constant development to obtain highly passivating TiO_x_ films [23]. Both Tetrakis (dimethylamido) titanium (TDMA-Ti) and titanium tetrachloride (TiCl_4_) precursors for TiO_x_ ALD are proven to provide high-quality TiO_x_ passivation layers for silicon solar cells [24,25,26]. The deposition of passivating TiO_x_ from the TiCl_4_ precursor was presented in the work of Yu et al. [25]. The minimum achieved surface recombination velocity was 44.24 cm/s for deposition at 200° C. The deposition temperature of TiO_x_ plays a crucial role, as documented by previous studies [27,28]. In 2021, Liu et al. achieved a high-performance passivating electron contact by deposition of aluminium oxide (Al_2_O_3_) and TiO_x_ in a stack using ALD at 150 °C [24]. With the application of TDMA-Ti and H_2_O precursors, a high minority carrier lifetime (τ_eff_) of 1.9 ms was obtained with a low contact resistivity of 0.1 Ω·cm^2^ [24]. Most ALD systems allow precise control of parameters such as deposition temperature [29], temperature of precursors [30], purging time [31], and number of deposition cycles. However, uniform control of the gases inside the reactor is not possible as the precursor gas distribution is controlled by the carrier gas. As such, large-scale industrial implementation of TiO_x_ ALD with a consistent product outcome is often challenging.

To approach consistent results, key parameters such as carrier gas flow, precursor pulse duration, purge duration, and deposition temperature must be optimised. In the current work, we present a comparison between two different precursors, namely TDMA-Ti and TiCl_4_, for ALD of TiO_x_ using different deposition and post-annealing process conditions. Furthermore, the effect of introducing aluminium oxide (AlO_y_) in the stack with TiO_x_ was investigated as aluminium oxide is widely applied not only as a passivation [23] layer but also as a tandem layer with other metal oxides [23,32] to improve the electronic properties of metal oxides such as resistance [33]. Electronic and crystalline properties of the deposited TiO_x_ and TiO_x_/AlO_y_ layers obtained in the current work were analysed using a range of techniques such as microwave photo-conductance decay (µ-PCD), four-point sheet resistance probe, and transmission electron microscopy (TEM).

## 2. Experimental Materials and Procedures

### 2.1. Material Preparation

Experimental samples were prepared using laser scribing of as-cut (100), n-type wafers into 3 × 3 cm size. The initial thickness and resistivity of the wafers were 180 µm and 1–3.5 Ω·cm, respectively.

An HNA solution (1HNO_3_ (75%):1CH_5_COOH (99.7%):0.2HF(45%)) was used for surface damage removal. Following damage removal, the samples were cleaned in an RCA 2 (0.1HCl (37%):0.2H_2_O_2_ (30%):H_2_O) solution at 70 °C. Next, the samples were immersed in a low-concentration HF solution for the removal of native oxide. The last part of the sample preparation was cleaning the samples in an RCA 1 (0.2NH_4_OH (30%):H_2_O_2_:H_2_O) solution at 70–75 °C (while forming so-called “RCA oxide”).

### 2.2. ALD Deposition

Atomic layer deposition of titanium oxide in this work was conducted from two different precursors TDMA-Ti and TiCl_4_. Two different ALD systems were used: Beneq TFS-500 for deposition of TiO_x_ using a TiCl_4_ precursor (at Aalto University, Helsinki, Finland) and Savannah S100 with TDMA-Ti as a precursor (at the Norwegian University of Science and Technology, Trondheim, Norway). In both cases, the second precursor was water. Each deposition set consisted of six samples, which, after the deposition, were split into three parallels of two samples. Post-deposition annealing (PDA) was performed in a rapid thermal annealing system (RTP Allwin). The second part of this study consisted of stack layer deposition of aluminium oxide (AlO_y_) and TiO_x_. Deposition of the AlOy in the AlO_y_/TiO_x_ stack was conducted using Trimethylaluminium (TMA) precursor as the first precursor and water as the second (on both ALD equipment Beneq TFS-500 and Savannah S100). The AlO_y_:TiO_x_ deposition ratios were: 1:1, 1:5, 1:30, and 1:60, respectively. All experimental details are presented in Table 1.

**Figure 1:**
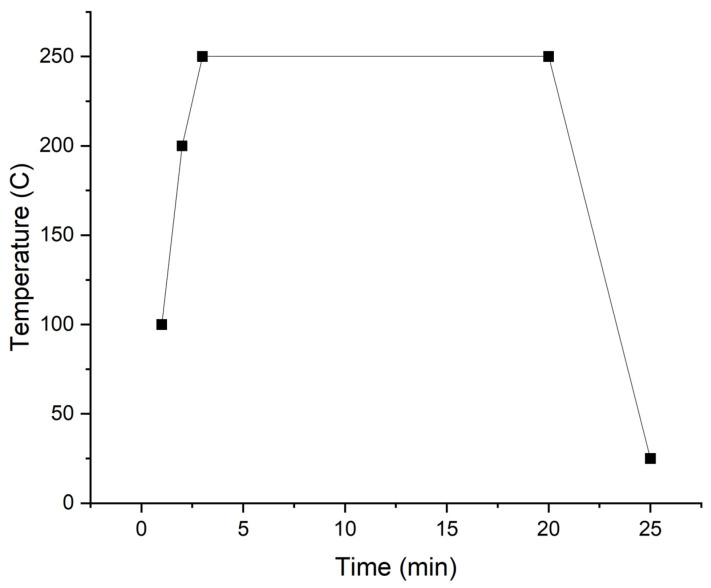
RTP system post-deposition annealing profile with a plateau of 250 °C.

Post-deposition annealing of the samples with the RTP system was conducted according to the RTP profile demonstrated in Figure 1 with the plateau temperatures outlined in Table 1.

### 2.3. Characterisation

After deposition and post-deposition annealing, the passivation and material properties of the thin films were studied. Minority carrier lifetime (MCLT) was measured using the transient photo-conductance decay (PCD) method with a Sinton WCT-120 tool, and the sheet resistance of the samples was determined using a CMT-SR2000N four-point probe. Five points per sample were measured using the four-point probe, and median values were calculated. Three samples per deposition condition such as thickness and deposition temperature were analysed. The samples with the highest MCLT and sheet resistance for both precursors were studied using TEM (transmission electron microscopy). Preliminary film thickness measurements were carried out using spectroscopic ellipsometry (Woollam M2000) for the TDMA-Ti precursor deposition. The thickness of the deposited TiO_x_ films was also measured using TEM. The TEM analysis was performed with a Helios 5 plasma-focused ion beam (PFIB). Electron-deposited carbon and subsequently platinum were used as protection layers. Cross-section lift-out and thinning were performed using Xe ions, finishing with 2 kV ions. TEM/annular dark field scanning TEM (ADF-STEM) was performed using a double-corrected JEOL ARM-200F cold field emission microscope at 200 kV. Energy-dispersive X-ray spectroscopy (EDS) and electron energy loss spectroscopy (EELS) were performed using JEOL Centurio and GIF Quantum detectors. Scanning precession electron diffraction was performed with a JEOL 2100F microscope at 200 kV.

## 3. Results and Discussion

### 3.1. TiO_x_ Films

**Figure 2:**
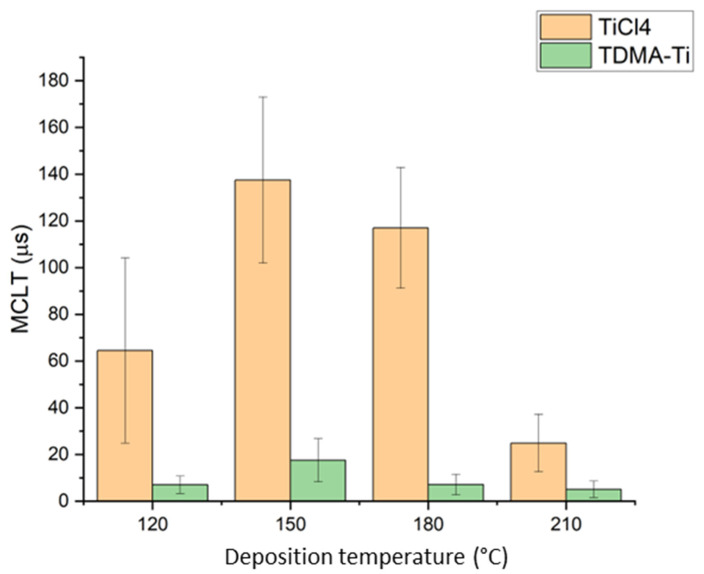
As-deposited median values of measured MCLT for an intended 15 nm thick TiO_x_ film deposited at different temperatures. Error bars illustrate the standard deviation for each condition/precursor.

The first part of this study concentrated on the deposition conditions for the two different TiO_x_ precursors. For both precursors, titanium oxide was deposited in the temperature range from 120 °C to 200 °C. During the experiments, an intended 15 nm thick TiO_x_ layer was deposited at these temperatures with subsequent µ-PCD measurements. Initial ellipsometry measurements showed a 1.5 nm average thickness error for the TDMA-Ti-deposited TiO_x_ films for the temperature range 120–200 °C (see Supplementary Material for details). Thus, the growth per cycle rate (GPC) was assumed to be constant for the investigated temperature range, in accordance with the previously reported value of 0.5 Å/cycle [34]. Figure 2 presents the measured values of the minority carrier lifetime for the as-deposited samples obtained from the TiCl_4_ and TDMA-Ti precursors, respectively. As seen in Figure 2, the TiO_x_ films deposited at 150 °C have the highest measured MCLT for both precursors and thus we conclude, in accordance with previous studies, that this temperature is most efficient in promoting the formation of the TiO_x_ anatase phase [35].

**Figure 3:**
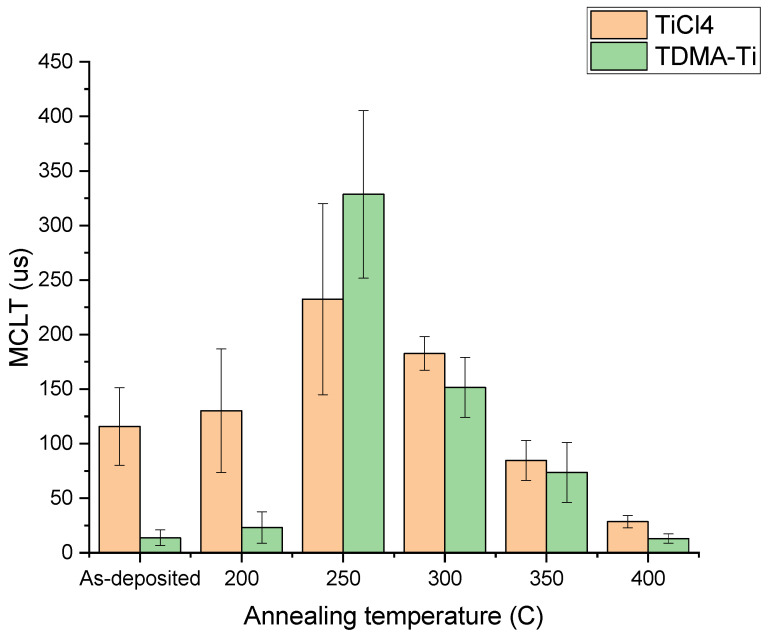
Measured median MCLT of the deposited 9 nm TiOx films deposited at 150 °C, post-annealed at different temperatures. Error bars illustrate the standard deviation for each condition/precursor.

The TiCl_4_ precursor films consistently display a higher minority carrier lifetime than the TDMA-Ti precursor. All MCLT results are, however, much lower than those presented in other studies [23,24]. This might be caused by the deposition recipe setup parameters or precursor distribution in the reactor. In some studies, the only reported results are obtained after post-deposition annealing, which has proven successful in increasing MCLT [24,26]. Thus, the samples deposited at 150 °C were annealed at 200 °C–400 °C for 20 min in N_2_ atmosphere with subsequent MCLT measurements. It was demonstrated that annealing at 250 °C significantly improves surface passivation for both precursors (Figure 3). The increase in MCLT for the annealed TiO_x_ deposited from the TDMA-Ti is larger than that of TiCl_4_, making the effect of the precursor on the MCLT after annealing insignificant at temperatures above 200 °C. Such a difference in the post-annealing MCLT improvement might be related to initial differences in structure, density, or thickness of the as-deposited films which, following annealing, become less significant. A more detailed structural analysis of the as-deposited films may provide additional information on the morphological differences between the as-deposited films from TiCl_4_ and TDMA-Ti. Additionally, we can see that annealing at or above 300 °C does not positively affect MCLT. Finally, it is possible that at temperatures over 250 °C, crystal nucleation is initiated at multiple sites in the oxide film, which results in phase transformation of TiO_x_ at these sites.

The thickness of the deposited layer is an important characteristic of the passivation- and carrier-selective layers. As such, TMOs (transparent metal oxides) typically display a clear correlation between the thickness of the deposited oxide and the passivation efficiency [36]. Thus, the relationship between thickness and passivation efficiency, as well as thickness and sheet resistance, were investigated for both precursors. TiO_x_ was deposited with an intended thickness range of 3–20 nm at 150 °C, with subsequent annealing. MCLT and sheet resistance were measured both before and after annealing.

**Figure 4:**
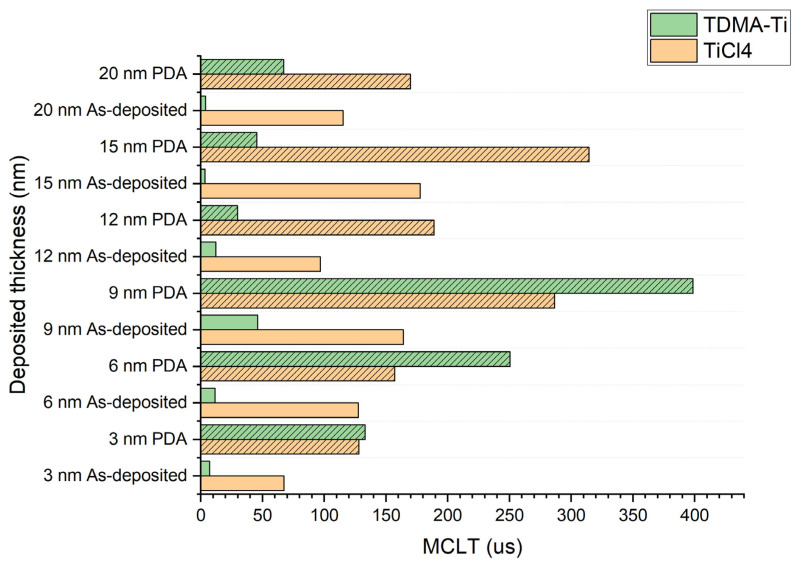
Measured median MCLT for the different deposited thicknesses of TiO_x_ before and after PDA.

Figure 4 presents the measured MCLT as a function of the targeted TiO_x_ thickness for TMDA-Ti and TiCl_4_ precursors for the samples deposited at 150 °C and annealed at 250 °C and for the non-annealed samples. In both cases, the passivation efficiency increased with TiO_x_ layer thickness up to 9 nm. Above 9 nm thickness, the passivation efficiency decreased for the TDMA-Ti precursor. The annealing treatment did not provide a significant improvement for 12, 15, or 20 nm of deposited TiO_x_ (TDMA-Ti). The reason for such poor response might be related to the thickness of the films. In the case of the reaction in the TiO_x_ layer or the reaction between the TiO_x_ layer and the Si/SiO_y_ interface, for the thicker oxide layers, a longer annealing time may be required. Thus, further experiments correlating the post-deposition annealing time with layer thickness may be needed.

**Figure 5:**
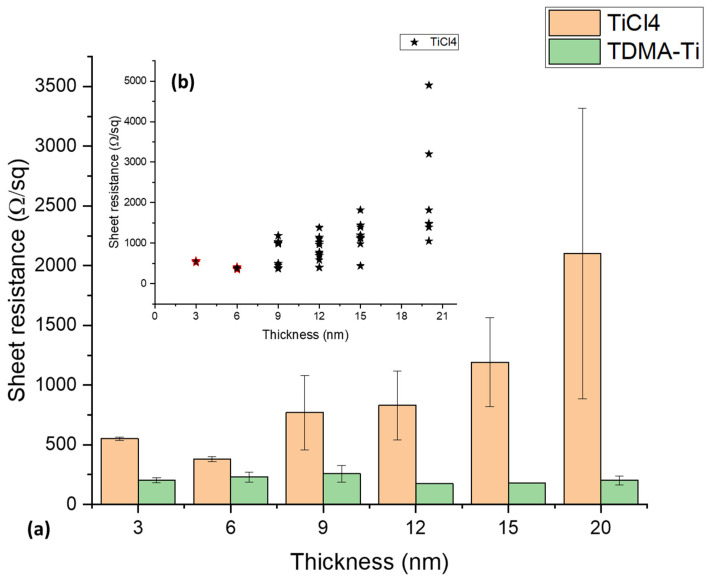
(**a**) Median sheet resistance measurement of the different thicknesses for the deposited TiO_x_ films after PDA. (**b**) Resistance measurement data distribution for the TiCl_4_ precursor. Error bars illustrate the standard deviations for each condition/precursor.

The sheet resistance of the TiO_x_ layers deposited at 150 °C and annealed at 250 °C was measured using the four-probe method. Figure 5a presents the measured sheet resistance for films deposited with both precursors at different thicknesses of the titanium oxide. We can observe a clear trend in resistance increase with thickness for the TiCl_4_ precursor while the resistance of the layers deposited from TDMA-Ti remains in the range of 170–250 Ω/sq. For the 20 nm thickness TiO_x_ deposited with the TiCl_4_ precursor, some measured resistance values are clear outliers (Figure 5b). Such abnormal values increase the median resistance and standard deviation. The low resistance of the TDMA-Ti (TiO_x_) might be due to a higher resistance-change threshold of the deposited TiOx caused by a change in the current path through the material. The TDMA-Ti-deposited TiO_x_ film resistance may increase faster after a certain thickness higher than 20 nm, as in other Ti-based materials [37,38]. However, to prove this theory, additional experimental work is required.

### 3.2. Al_2_O_3_/TiO_x_ Stack Films

Techniques using aluminium oxide in a stack with titanium oxide [39] or aluminium-doped TiO_x_ [24] are under development, mostly because of the improved conductivity of such layers in comparison to TiO_x_ alone. Such passivation layers are one of the alternatives to ultra-thin a-Si:H passivation [40]. In this work, AlO_y_/TiO_x_ stacks were studied as a possible alternative to mono-TiO_x_ layers, quantifying the potential improvement for each precursor.

**Figure 6:**
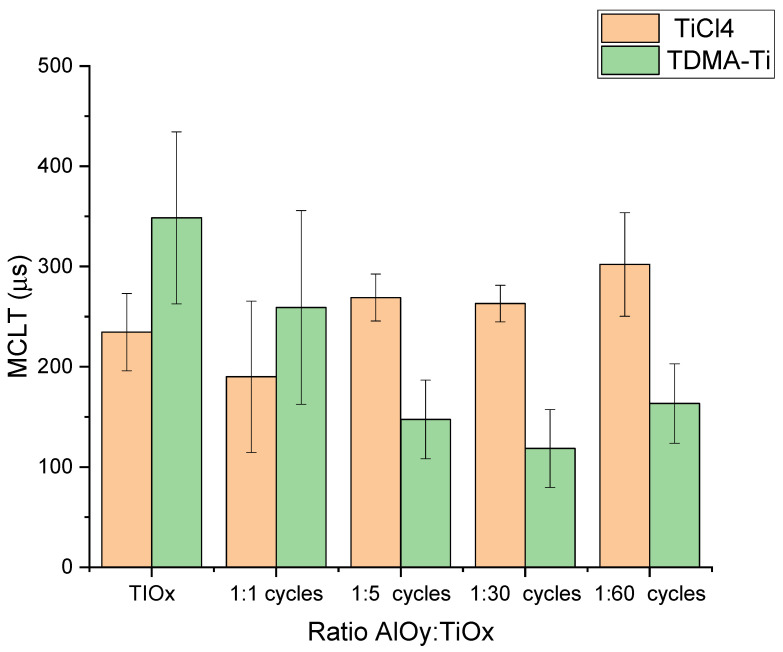
Measured median MCLT of the deposited AlO_y_/TiO_x_ stack depending on the AlO_y_:TiOx deposition cycle ratio after annealing at 300 °C. Error bars illustrate the standard deviations for each condition/precursor.

Figure 6 illustrates the effect of introducing aluminium oxide in the TiO_x_ stack (intended 9 nm) for both precursors. For neither precursor, there is clear improvement in MCLT when introducing AlO_y_ in the stack.

**Figure 7:**
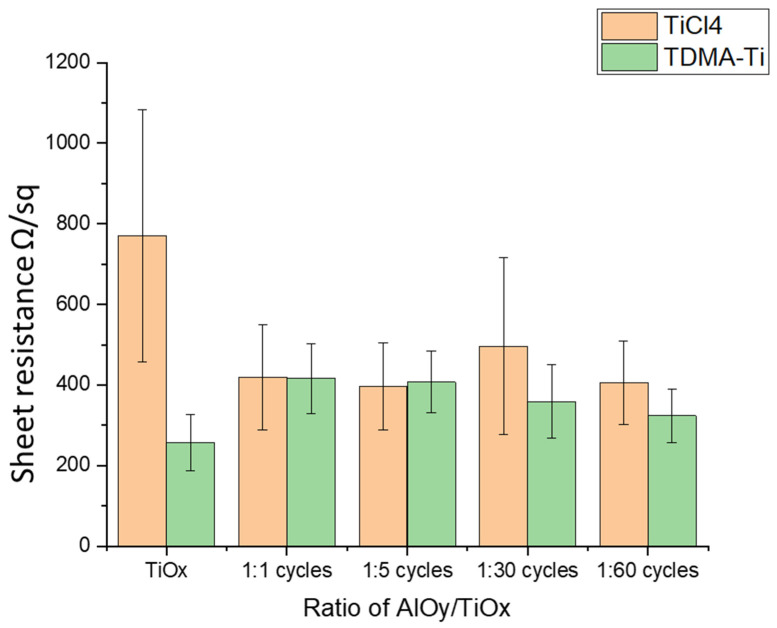
Measured median sheet resistance of the deposited AlO_y_/TiO_x_ stack depending on the AlO_y_:TiOx deposition cycle ratio after annealing at 300 °C. Error bars illustrate the standard deviation for each condition/precursor.

Following the MCLT analysis of the samples, the sheet resistance of the Al_2_O_3_/TiO_x_ stacks was measured. In Figure 7, no clear differences in resistance between the precursor stacks were observed. However, resistance was reduced by more than 40% (770 Ω/sq to 405 Ω/sq) from the original values of the TiO_x_ layer for the TICl_4_ precursor. The measured sheet resistance of the deposited AlO_y_/TiO_x_ stacks was at approximately the same level for all AlO_y_/TiO_x_ ratios. The measured sheet resistances are detailed in the Supplementary Materials.

### 3.3. TEM Analysis of Deposited Films

In order to better understand the differences observed between precursors, a TEM analysis of three samples for each of the two titanium oxide ALD precursors was carried out. These included the targeted 9 nm of pure TiO_x_ annealed and as-deposited, along with the targeted 9 nm deposited AlO_y_/TiO_x_ stacks.

**Figure 8:**
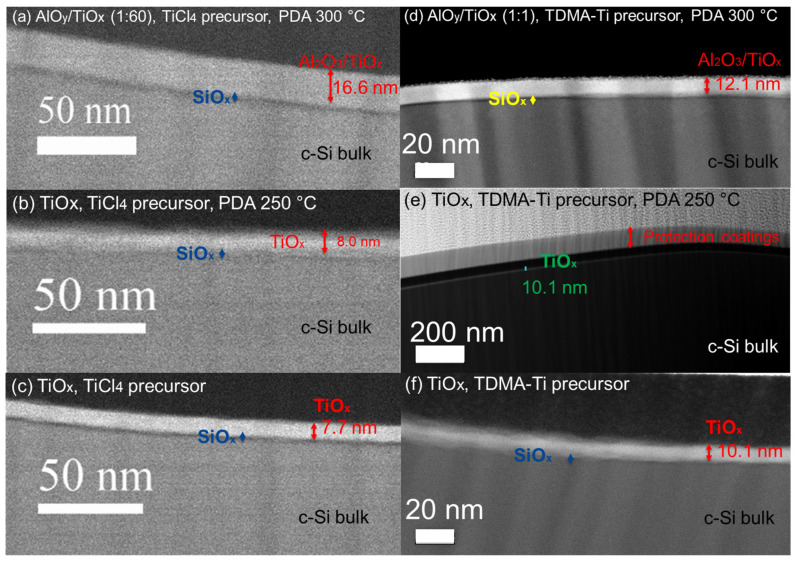
ADF-STEM image showing the TiOx and AlO_y_/TiO_x_ stack layers: (**a**) Annealed at 300 °C AlO_y_/TiO_x_ stack (1:60), TiCl_4_ precursor, (**b**) annealed at 250 °C TiO_x_ layer, TiCl_4_ precursor, (**c**) TiO_x_ as-deposited, TiCl_4_ precursor, (**d**) annealed at 300 °C AlO_y_/TiO_x_ stack (1:1), TDMA-Ti precursor, (**e**) annealed at 250 °C TiO_x_ layer, TDMA-Ti precursor, and (**f**) TiO_x_ as-deposited, TDMA-Ti precursor.

First the achieved, as opposed to targeted, TiO_x_ and AlO_y_/TiO_x_ layer thicknesses were measured. The actual deposited thickness of the targeted 9 nm layers for the TiCl_4_ precursor was 16.6 nm for the annealed AlO_y_/TiOx (1:60) stack (Figure 8a), while for the annealed TiO_x_ (Figure 8b) and as-deposited TiO_x_ (Figure 8c), the thicknesses were 8 nm and 7.7 nm, respectively. For the TDMA-Ti precursor, the thickness was 12.1 nm for the annealed AlO_y_/TiO_x_ (1:1) stack (Figure 8d), 10.1 nm for the as-deposited TiO_x_ (Figure 8f), and 10.1 nm for the annealed TiO_x_ (Figure 8e). As such, the intended deposited thicknesses and the actual thickness of the layers were slightly different for the single TiO_x_ deposited films. The thickness of the AlO_y_/TiOx (1:60) stack using the TiCl_4_ precursor was 7.6 nm thicker than expected, while the TDMA-Ti precursor thickness of the AlO_y_/TiO_x_ (1:1) stack was 3.1 nm thicker than intended. However, the pure TiO_x_ layers deposited using the TiCl_4_ precursors are somewhat thinner than intended, while those deposited using TDMA-Ti are slightly thicker than expected.

As the initial ellipsometry measurements showed small thickness deviations for the TDMA-Ti-deposited TiO_x_ films, these measurements were not continued for the bulk of samples. Hence, there may be differences in the thicknesses measured using ellipsometry and those measured using TEM. Further work to compare the thicknesses obtained using TEM and ellipsometry should hence be performed in future studies.

The TEM-measured thicknesses of the AlO_y_/TiO_x_ stacks also deviate from those intended. During the deposition, precursors were pulsed into the reactor one by one after a certain amount of time (purging time). It is possible that the purging time for AlO_y_ was not long enough to remove the products of the TMA and H_2_O precursor reaction [41], which resulted in the additional growth of the AlO_y_ layer during the next pulses of water precursor into the reactor. Thus, during the deposition of TiO_x_ and AlO_y_, additional TMA precursors may remain in the reactor, resulting in the additional growth of the oxide layer.

**Figure 9:**
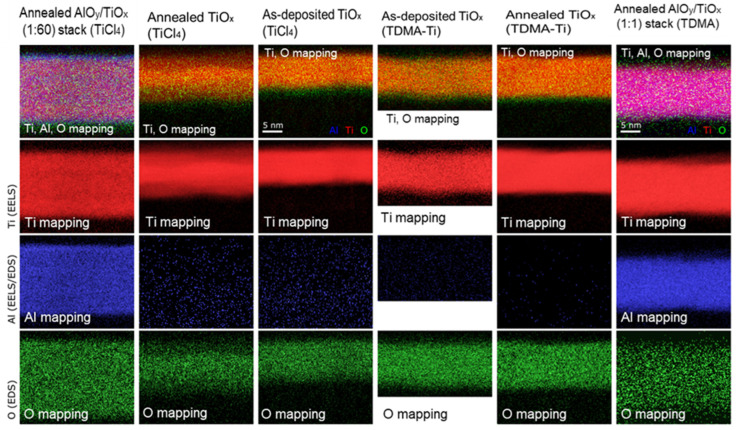
EDS/EELS elemental mapping of the samples analysed with TEM.

Elemental mapping using a combination of electron energy loss spectroscopy (EELS) and energy-dispersive X-ray spectroscopy (EDS) was also carried out for each of the samples (Figure 9). While EDS is not a quantitatively reliable tool, it gives a good indication of the relative concentration of elements. A comparison of the AlO_y_/TiO_x_-deposited stacks revealed that Al is distributed across the whole oxide layer for both precursors, while the deposition process was performed layer-by-layer. In the case of the 1:1 AlO_y_/TiO_x_ ratio, such an Al distribution might be possible due to the proposed TDMA-Ti precursor residue in the reactor. In the case of the 1:60 ratio, where only 1 deposition cycle of AlO_y_ was performed for 60 deposition cycles of the TiO_x_, and each cycle had its own purging, the probability of the AlO_y_ being distributed across the whole oxide layer caused by TDMA-Ti precursor residues is low. However, the thickness of the deposited layer per cycle is approximately 0.05 nm for TiO_x_, while it is 0.1 nm for the AlO_y_ [34]. As a result, for the 1:1 cycle deposition, the thin film will consist of 0.05–0.1 nm thick layers, which are not possible to identify with the resolution of the TEM instrument used in this work. Thus, the conclusion is that the EDS analysis does not have a high enough resolution to give an accurate composition of the individual deposited layers.

For both the as-deposited and annealed TiO_x_ layers, a difference in the Ti:O ratio between precursors was found. For both precursors, there was also a difference between the as-deposited and annealed TiO_x_, which corresponded with the layer thickness differences. The composition of the TiO_x_ films from elemental mapping measured in atomic percentage using EDS analysis is presented in Table 2. Although the values obtained using EDS cannot be claimed to be quantitatively exact, relative differences are more reliable.

As summarised in Table 2 and suggested by the MCLT measurement results, it is possible that the oxygen to titanium content dictates passivation properties. We can see that the annealed samples of both precursors display a higher oxygen-to-titanium ratio and a higher minority carrier lifetime than the as-deposited samples. This phenomenon was also observed in previous work for the E-beam evaporated TiO_x_ layer [42], indicating that a higher oxygen content improves the passivation properties of the TiO_x_ layer.

The TEM work revealed that the TDMA-Ti-deposited TiO_x_ was 2 nm thicker than the TiCl_4_-based film. Such a thickness difference might affect the MCLT, as passivation tends to increase with the thickness of the oxide layer [43,44]. The same trend is presented in this work for the annealed TiO_x_ from 3 to 9 nm thickness (Figure 2). This theory would also explain the higher MCLT of the TDMA-Ti-deposited TiO_x_ layer (at thicknesses ≤ 9 nm intended thickness) over the TiCl_4_ precursor. However, if we assume that each deposited TiO_x_ TDMA-Ti thickness is 2 nm thicker than intended, in accordance with Figure 8, the 9 nm thick layer of TDMA-Ti TiO_x_ still has a relatively higher MCLT than the 12 nm thick TiCl_4_-based TiO_x_ film.

**Figure 10:**
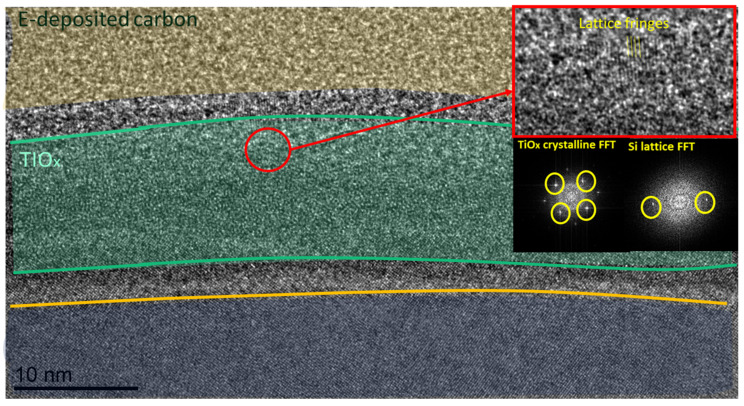
High-resolution TEM image showing the annealed TiO_x_ sample (TiCl_4_ precursor) with Fourier transforms (FFTs) of the layer attached (FFT zone axis (001)).

**Figure 11:**
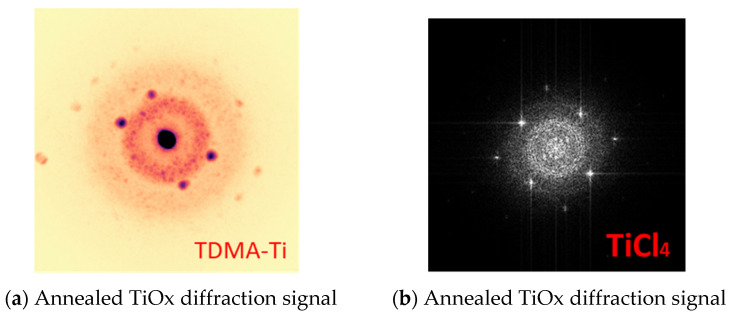

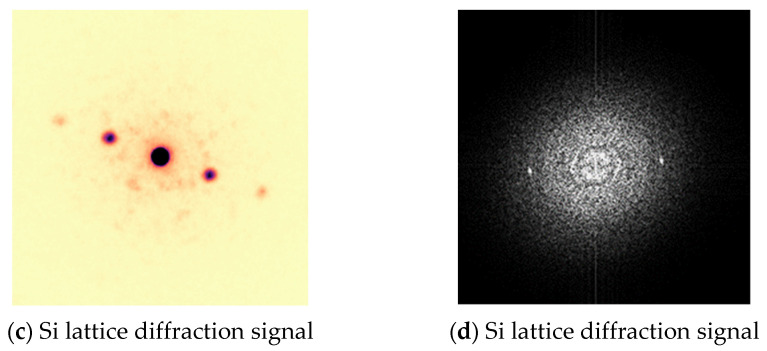
Diffraction peaks from the selected area electron diffraction scanning for TiO_x_ deposited with different precursors.

During the high-resolution TEM analysis of the annealed samples obtained from the TiCl_4_ precursor, crystallised areas were discovered. Figure 10 shows a high-resolution TEM image of annealed TiO_x_ (TiCl_4_) film (8 nm) with faint signals of crystallinity. For neither of the AlO_y_/TiO_x_ stacks (TiCl_4_) nor the as-deposited TiO_x_ (TiCl_4_) samples, lattice diffraction was indicated. For the annealed TDMA-Ti precursor samples, the same signals of crystallinity were also revealed with electron diffraction of the selected area. Figure 11 shows the diffraction patterns in the annealed TiO_x_ for both precursors. Like the TiCl_4_ precursor, the as-deposited samples from TDMA-Ti showed no traces of crystallinity.

Signs of nano-crystallinity for annealed TiO_x_ have been reported in previous studies. Phase-change from amorphous TiO_x_ to anatase in atomic-layer-deposited TiO_x_ (from titanium IV isopropoxide TTIP precursor) was discovered in a post-deposition annealing process at 450 °C [45]. In our case, the temperature of the PDA process was lower at 250 °C, and the crystallinity signal was weak. However, this may indicate that already at 250 °C, the phase-change process is initiated in ultra-thin TiO_x_ films. In order to gain valuable information on the crystallinity of TiO_x_ films, conducting grazing incidence X-ray diffraction analysis must be considered in future research.

Crystallinity might affect passivation quality. During the TiO_x_ phase change process, additional oxygen might be absorbed, which will decrease oxygen deficiency in the film. Another possible reason for the improvement in the measured MCLT after the PDA process is the additional growth of the silicon oxide (SiO_2_) at the Si/TiO_x_ interface [42,46]. Previous studies have presented opposite effects on passivation quality with the crystallisation of TiO_x_ [46]. However, a previous study by the current first author on E-beam-deposited TiO_x_ indicated the same passivation improvement with film crystallisation [42].

Neither of the annealed AlO_y_/TiO_x_ films deposited from TiCl_4_ and TDMA-Ti precursors showed signs of crystallinity despite a higher annealing temperature (300 °C). These samples also showed less improvement in passivation quality after the PDA process. This might be related to a too-low temperature for “activation” of AlO_y_ passivation [47] in conjunction with the absence of a real stack, as aluminium is distributed across the whole film. This probably affected the sheet resistance of the mixed oxide layer in comparison with TiO_x_ alone, as Al is widely used as a dopant to improve the conductivity of MO films [24,48]. The aluminium oxide presence might also hinder the crystal formation (phase change) of TiO_x_. The reason for such an effect might be the formation of a new aluminium/titanium compound (Al_x_-O_y_-Ti_z_) in the passivation layer, resulting in the distribution of Al across the oxide layer. Thus, a different temperature is required to improve the passivation quality of the layer as well as initiate a phase change process for TiO_x_.

## 4. Conclusions

This work aimed at comparing titanium oxide precursors (TiCl_4_ and TDMA-Ti) for atomic layer deposition of passivating TiO_x_. The precursors were compared with respect to the deposition temperature, post-deposition annealing composition, and properties of deposited films.

A deposition temperature of 150 °C was found to be optimal for both precursors. It was confirmed that post-deposition annealing of the deposited TiO_x_ improves the passivation quality of the oxides. The optimal post-deposition annealing temperature of 250 °C was also similar for the two precursors.

The maximum achieved minority carrier lifetime (τ_eff_) for the deposited 9 nm of TiO_x_ was 398 µs for the TDMA-Ti precursor and 286 µs for TiCl_4_. A TEM analysis of the as-deposited and annealed TiOx revealed signals of nano-crystallinity for the post-deposition annealed TiO_x_ films for both precursors, indicating that a phase change of the atomic-layer-deposited TiO_x_ possibly starts at 250 °C. However, there is no evidence of a correlation between crystallinity and passivation improvement.

Aluminium oxide (AlO_y_)/titanium oxide (TiO_x_) stacks showed higher passivation quality than single TiO_x_ films for both precursors with a maximum (τ_eff_) of 311 µs for the TiCl_4_ precursor. For the TiCl_4_ precursor, the passivation quality increased with a decreasing AlO_y_:TiO_x_ cycle ratio (from 1:1 to 1:60), while for the TDMA-Ti precursor, it was vice versa (from 1:60 to 1:1). Furthermore, sheet resistance measurements showed that the AlO_y_/TiO_x_ stack had much lower resistance than the pure TiO_x_ layer. Thus, we conclude that the Al presence in TiO_x_ films decreases sheet resistance, while additional experimental work, such as optimising annealing temperature, is required to improve the passivation quality of the AlO_y_/TiO_x_ stack.

## 5. Future Perspectives

In order to continue the work of the current study, some future perspectives may be presented: The performance of the deposition process for the two different ALD equipment units should be tuned to the same GPC for the two precursors and, if possible, also ensure the same precursor distribution during the deposition. This might be helpful to better evaluate the correlation between deposited thickness and differences in sample characteristics (e.g., MCLT). Moreover, further post-deposition analysis of the obtained films such as grazing incidence X-ray diffraction analysis and transfer length measurements (TLM) will give a better understanding of film conductivity and crystallinity. Further experimental work investigating the effect of post-annealing duration on different TiO_x_ film thicknesses is recommended.

## Figures and Tables

**Table 1:** Main experimental conditions.

Deposited Thickness of TiOx (nm)	3, 6, 9,12, 15, 20
Deposition temperature of TiOx from TDMA-Ti (°C)	120, 140, 150, 180, 200, 210
Post deposition annealing temperature (°C)	200, 250, 300, 350, 400
Deposition temperature of TiO_x_ from TiCl_4_ precursor (°C)	120, 150, 180, 210
Deposition layer stack ration AlO_y_:TIO_x_	1:1, 1:5, 1:30, 1:60

**Table 2:** Atomic percentage of the TEM EDS-analysed samples. Atomic percentage error indicates summed EDS fitting error during a measurement.

	Ti, Atomic %	O, Atomic %
As-deposited TiO_x_ (TiCl_4_)	22.83 ± 0.25	77.17 ± 0.78
As-deposited TiO_x_ (TDMA-Ti)	29.53 ± 0.21	70.47 ± 0.47
Annealed TiO_x_ (TiCl_4_)	20.76 ± 0.31	79.24 ± 0.99
Annealed TiO_x_ (TDMA-Ti)	26.09 ± 0.20	73.80 ± 0.48

## Data Availability

Available on request to authors.

## Supplementary Materials

The following supporting information can be downloaded at: https://www.mdpi.com/article/10.3390/ma16165522/s1.
