# Supplementary material for: Atomic Layer Deposition of Titanium Oxide-Based Films for Semiconductor Applications—Effects of Precursor and Operating Conditions
Source: Materials (Basel). 2023 Aug 8;16(16):5522. doi: 10.3390/ma16165522 (PMC10456286; doi:10.3390/ma16165522)
Supplement: Supplementary file 1 [file materials-16-05522-s001.zip › materials-2490911-supplementary.pdf]

Table S1. *Measured sheet resistan*

Precursor	Thickness, nm	point1	point2	point3	point4	point5	Ω/sq
TDMA-Ti	3	222.7	242.5	220.2	204.1222		
TDMA-Ti	3	207.8	185.9	171.8			
TDMA-Ti	3	177.9	206.4	201.9			
TDMA-Ti	6	171.5	281.5	238.6	230.5111		
TDMA-Ti	6	271.3	236.1	169			
`TDMA-Ti	6	269	182.8	254.8			
TDMA-Ti	9	291.4	404.1	288.4	257.8444		
TDMA-Ti	9	300.8	193.4	275			
TDMA-Ti	9	194.1	195.4	178			
TDMA-Ti	12	174	176.2	173.7	174.9444		
TDMA-Ti	12	173.6	173.3	173.3			
TDMA-Ti	12	178.8	178.4	173.2			
TDMA-Ti	15	175	180	175.6	179.1667		
TDMA-Ti	15	179.6	178.2	181.2			
TDMA-Ti	15	180	182	180.9			
TDMA-Ti	20	171.4	181	174.5	201.3444		
TDMA-Ti	20	242.6	272.3	236			
TDMA-Ti	20		175.8	174.9	183.6		
$TiCl_4$	3	569.5	555.5	535.4	546.7		
$TiCl_4$	3	554	557.5	524.9	567.8		
$TiCl_4$	6	375.5	6.557439	384	375	368	
$TiCl_4$	6	390	18.28934	389	402	404	
$TiCl_4$	6	378.5	30.68659	347	420	368	
$TiCl_4$	9	345.6	349.3	285.1	455.2		
$TiCl_4$	9	405.6	447.1	516	403.6		
$TiCl_4$	9	661.8	1073	1032	1180		
$TiCl_4$	12	976.45	151.7371	1019	967.8	1142	
$TiCl_4$	12	519.75	143.9685	406	692	396	

TiCl$_4$	*12*	*1008.2*	*266.6725*	*736*	*1384*	*768*	
TiCl$_4$	*15*	*1818*	*1395*	*1116*	*1197*		
TiCl$_4$	*15*	*1252.8*	*106.1965*	*1415*	*1248*		
TiCl$_4$	*15*	*1220*	*1261*				
TiCl$_4$	*20*	*3201*	*1476*	*4896*	*1817*		
TiCl$_4$	*20*	*1393*	*1483*	*1053*	*1488*		

ce depending on the TiOx film thickness and used deposition precursor

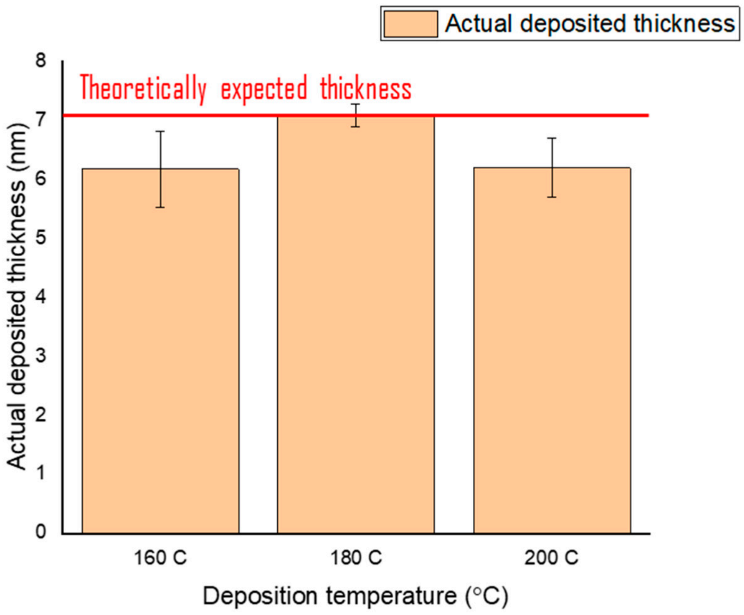

Figure S1. *Ellipsometry measurements of the deposited TiOx film at different temperatures for TDMA-Ti precursor (140 deposition cycles)*
